# County-scale dataset indicating the effects of disasters on crops in Taiwan from 2003 to 2022

**DOI:** 10.1038/s41597-024-03053-1

**Published:** 2024-02-14

**Authors:** Yuan-Chih Su, Yuan Shen, Chun-Yi Wu, Bo-Jein Kuo

**Affiliations:** 1 Department of Agronomy, National Chung Hsing University, Crop Science Building, No. 145, Xingda Road, South District, Taichung City, 40227 Taiwan (R.O.C.); 2 International Master Program of Agriculture, National Chung Hsing University, No. 145, Xingda Road, South District, Taichung City, 40227 Taiwan (R.O.C.); 3 Department of Soil and Water Conservation, National Chung Hsing University, No. 145, Xingda Road, South District, Taichung City, 40227 Taiwan (R.O.C.); 4 Smart Sustainable New Agriculture Research Center (SMARTer), Taichung City, 40227 Taiwan (R.O.C.)

**Keywords:** Natural hazards, Agroecology

## Abstract

A lack of crop disaster datasets has limited the exploration of the influence of small-scale disasters on crops. Because disasters are often defined on the basis of human impact, disaster databases may underestimate the effect of disasters on crop production. Additionally, the resolution of such databases is insufficient for evaluating the effects of disasters on small areas. In this study, crop disaster and daily weather datasets covering the period from 2003 to 2022 in Taiwan were developed. Total 9,245 damage records from 233 observations of crop disasters were mined from the Report on Crop Production Loss Caused by Disasters of Taiwan. Daily weather data were collected from weather stations. Entire crop disaster information including multiple disasters, crops, and affected regions was stored in crop disaster dataset. All datasets were cleaned up and refined to enhance their quality, and characteristics such as disaster and crop classification were added to enhance the applicability of these datasets. These datasets can be used to determine the relationship between disaster type and crop production losses.

## Background & Summary

Because of climate change, many studies have focused on the effects of natural disasters, such as extreme temperatures, drought, and heavy rainfall, on crops^1–3^. Such studies have evaluated the effects of disasters on crop production by relating weather information to crop yield data. However, accurate data with well-defined crop disasters are required to provide an effective evaluation of these effects, particularly for small areas.

Environmental factors, including natural disasters, influence crop production^4^. According to the Food and Agriculture Organization (FAO) of the United Nations, between 2008 and 2018, Asia experienced larger losses in crop and livestock production as a result of natural disasters compared with Africa, Latin America and the Caribbean, Northern America, Europe, and Oceania^4^; the losses in Asia accounted for 74% of the total global losses (US$ 280 billion). Floods and storms are the primary sources of disasters in Asia. Studies have examined the increasing frequency of natural disasters within the context of climate change^5–8^. This increase in disasters must be properly and rapidly addressed to prevent adverse effects on crops.

Disaster databases are an effective tool for studying the characteristics and trends of disasters, and they can be used to mitigate risks and develop adaptation strategies on both the national and global scales^9^. Disaster loss databases are valuable for evaluating risk for insurance purposes and conducting socioeconomic analyses for decision-making^10^. Multiple national and international disaster databases—such as the Emergency Event Database (EM-DAT), NatCatSERVICE Database (Munich Re), and SIGMA (Swiss Re)—have been established for research purposes^10–12^. In addition, the GLobal IDEntifier (GLIDE) database (https://glidenumber.net/glide/public/search/search.jsp) has been developed by the Asian Disaster Reduction Center to address problems with data scattering and unclear disaster information. The GLIDE database contains data on disasters, each of which is assigned a unique ID for identification. The database is shared and promoted by various institutions, such as the FAO, World Bank, and European Commission. Nevertheless, certain disasters affect only limited areas, and the aforementioned databases cover few localized or small-scale disasters, which can affect crop production as much as large disasters^4,10^. Therefore, results obtained using data from national and regional databases may not be applicable to small areas and may underrepresent the effects of natural disasters^13,14^. In addition, the aforementioned databases cover disasters that affect only human impact. For example, the EM-DAT covers disasters meeting at least one of the following criteria: (i) causing 10 or more human deaths, (ii) rendering 100 or more individuals injured or homeless, (iii) requiring a national declaration of a state of emergency, and (iv) eliciting international assistance. Therefore, the data from disasters that affect only crop production are not included in the aforementioned databases. To adapt to climate change and develop disaster risk mitigation strategies to protect crop production, the effects of small-scale or “silent” disasters, which are often unreported but harmful to the livelihoods of farmers, must first be understood^4^. Because data on small-scale disasters are typically disseminated through different sources, such as news reports and yearbooks, these data are difficult to systematically collect. Therefore, we developed a county-scale crop disaster dataset containing data methodically collected by the government on various types of disasters and their effects on crops.

Taiwan is an island in East Asia, located in the northwestern Pacific Ocean. Over two-thirds of Taiwan’s terrain is mountainous, particularly that in the east. Mountain ranges run from north-northeast to south-southwest, roughly parallel to the east coast. As a result of Taiwan’s location and geography, its climate ranges from tropical to subtropical and tends to be temperate in the mountainous regions. This climate variation allows for crop diversity. However, every year, various natural disasters result in crop production losses. Because Taiwan is situated between two major typhoon paths^15^, typhoons the primary disasters affecting the island. In addition to typhoons, extreme rainfall caused by the mei-yu front affects crop production^16^. As a result of these natural disasters, crop production losses amounting to an average of US$ 318 million were observed in Taiwan from 2012 to 2021^17^. In 1991, the Agricultural Natural Disaster Relief Act (ANDRA) was established to ensure the economic well-being of Taiwanese farmers after disasters. Within the framework of the ANDRA, farmers are provided with compensation for disaster-induced losses. Before such compensation is provided, the extent of crop damage is evaluated by agricultural technicians from the relevant district office. The Ministry of Agriculture (MoA, formerly the Council of Agriculture, renamed in August 2023) collects records of disaster-induced crop damage and releases a report. Because of Taiwan’s geographic location and climate characteristics, crop disaster data collected in Taiwan can provide useful information to other Asian countries to employ in risk assessment, especially for predicting effects of typhoons and extreme rainfall on small farms. In addition, the diversity of crop species in Taiwan means that studies of Taiwanese crops can be helpful for researchers interested in the effects of disasters on various crops. In this study, we refined data from the Report on Crop Production Loss Caused by Disasters of Taiwan (hereafter, the Report) from 2003 to 2022 to establish a crop disaster dataset. Data from this report have previously been used to elucidate the relationships between disasters and crop production losses^18–20^. Extreme wind speed and heavy rainfall result in losses of grain and vegetable crops^18^. Our dataset includes different types and scales of disasters with corresponding crop damage records. This dataset can be used to predict the effects of natural disasters on crops, evaluate risks for crop insurers, and provide information for agricultural decision-making. To further understand crop production losses caused by different weather conditions, we also developed a daily weather dataset to describe the weather during each crop-damaging disaster.

## Methods

The Python was used to conduct web crawler for downloading the daily meteorological data from staffed and automated weather stations, and automated rain gauge stations. All raw data including weather dataset and disaster dataset were proceed and cleaned separately by SAS version 9.4 for Windows (SAS Institute, Cary, NC, USA).

### Crop disaster dataset processing

After a disaster, agricultural technicians from relevant district offices investigate and report on crop production losses to the local governments (municipality, county, and city). Depending on the extent of such losses, each local government requests assistance, that is, disaster relief, from the central government. After inspection and approval, the central government announces the object (county and crop) of relief. To obtain subsidies, farmers submit their applications to the district office. Subsequently, agricultural technicians inspect each application to evaluate crop losses, and they collect relevant information—such as the area of each farmer’s field (damaged field area), the proportion of crops destroyed in the field (damage level), and the type of crop—to generate a detailed report of crop production losses. These reports are collected by the local government. Finally, a report is released annually by the central government.

Crop loss records contain data pertaining to the affected location, disaster type, typhoon name, disaster year, disaster date, affected crop, damaged field area (ha), damage level (%), actual damaged area (ha), estimated production loss (tonnes), and estimated value loss (new Taiwan dollars, NTD). In this study, data were collected from the 2003 to 2022 editions of the Report. Report files were downloaded from the MoA website (https://agrstat.moa.gov.tw/sdweb/public/official/OfficialInformation.aspx) and converted into CSV format. Data were extracted from each damaged record to establish a crop disaster dataset. Damaged field area, damage level, actual damaged area, estimated production loss, and estimated value loss were used to indicate the effects of natural disasters on crop production; these are described as impact variables. Here, an affected location is a county affected by a disaster. Given the size of the study area, the data obtained from Chiayi, Hsinchu, and Taipei City were combined with those obtained from their respective counties (Taipei County became New Taipei City in 2010). Similarly, the data obtained from Taichung, Tainan, and Taoyuan City were combined with those obtained from their respective counties because these administrative divisions were officially merged due to the reorganization of counties. In addition, the data obtained from Keelung City were combined with those obtained from Taipei County/New Taipei City. If a disaster involved a typhoon, the typhoon’s name was included. Damaged field area was defined as the total field area dedicated to each crop affected by a disaster in one county. Damage level was defined as the average percentage of field area damaged by a disaster such that harvest was impossible. Actual damaged area was calculated as damaged field area times damage level. The average local (district) yield of each crop was multiplied by the actual damaged area for estimating the crop production loss. The estimated production loss was multiplied by the market price of each crop (NTD kg^−1^) to estimate value loss.

To refine the crop disaster dataset, some variables were adjusted, and new variables were added. A disaster number was generated on the basis of its year (four digits) and a sequential number (three digits) for the disaster within the year. The affected counties were grouped into four regions: central, eastern, northern, and southern regions. Because years in the Report are based on the Republic of China calendar, these were converted to Gregorian years through the addition of 1911. In the report, disaster dates are expressed using a specific date or a broad period e.g., mid-July or May). For typhoons and certain heavy rainfall disasters, the disaster period was refined using data obtained from the National Science and Technology Center for Disaster Reduction (https://den.ncdr.nat.gov.tw/). After adjustment, disaster periods were expressed in terms of a start date and end date. In addition, estimated value loss was converted into units of US$ 1,000. The type, group, and class of damaged crops were defined in the disaster dataset in accordance with the classification of the FAO (Indicative Crop Classification, version 1.1)^21^, which is based on the product type, crop genus or species, and crop type (temporary or permanent). The type of crop was used to distinguish between temporary and permanent crops. The crops were initially classified into groups, such as cereals, vegetables, and fruits. Each group was subsequently divided into classes, such as leaf or stem vegetables and citrus fruits. Damaged crops that could not be classified were assigned a blank classification. The botanical name of each crop was used to specifically define it. In addition, the group, subgroup, main type, subtype, and sub-subtype were used to classify disasters in accordance with the definitions and classifications of the EM-DAT. Disaster group was used to differentiate natural and technological disasters. Disaster subgroup was used to differentiate biological, geophysical, climatological, hydrological, meteorological, and extraterrestrial natural disasters. Main disaster types, including storms and extreme temperatures, were broken down into disaster subtype. Table 1 summarizes different crop disasters and their corresponding main types, subtypes, and sub-subtypes. Because some crop damage records contained data on secondary disasters, subgroup, main type, disaster subtype, and sub-subtype were numbered 1 and 2.

**Table 1:** List of crop disasters and disaster type classification in the raw crop disaster dataset.

Crop Disasters	Disaster Main Types	Disaster Sub-Types	Disaster Sub-Sub-Types
Cold wave	Extreme temperature	Cold wave	
Continuous rain	Storm	Convective storm	Rain
Drought	Drought		
Earthquake	Earthquake	Ground movement	
Extremely heavy rain	Storm	Convective storm	Rain
Foehn			
Front	Extreme temperature	Cold wave	
Frost	Extreme temperature	Severe winter conditions	Frost
Grafting pear damage			
Gust wind	Storm	Convective storm	Wind
Hail	Storm	Convective storm	Hail
Heavy rain	Storm	Convective storm	Rain
High temperature	Extreme temperature	Heat wave	
Low temperature	Extreme temperature	Cold wave	
Pest			
Rain damage	Storm	Convective storm	Rain
Southwesterly flow	Storm	Convective storm	Rain
Strong wind	Storm	Convective storm	Wind
Thunderstorm	Storm	Convective storm	Thunderstorm
Tornado	Storm	Convective storm	Tornado
Tropical depression	Storm	Tropical storm	
Typhoon	Storm	Tropical storm	
Unusual climate			
Unusual wind	Storm	Convective storm	Wind

### Daily weather dataset processing

In this study, daily meteorological data were collected from staffed and automated weather stations, automated rain gauge stations, and agricultural weather stations operated by the Central Weather Administration (CWA, formerly the Central Weather Bureau, renamed in August 2023) of Taiwan (for more information, please visit https://codis.cwa.gov.tw or https://agr.cwa.gov.tw). These automatic rain gauge stations measure only daily precipitation. Data were obtained from 580 weather stations and 307 rain gauge stations. The daily weather data included were the mean air temperature (°C), maximum air temperature (°C), minimum air temperature (°C), average relative humidity (%), mean wind speed (m s^−1^), mean wind direction (°), total precipitation (mm), total radiation (MJ m^−2^), total sunshine hours (h), and total evaporation (mm). The daily weather dataset included details about each station, such as the station code, altitude, longitude, latitude, location, and region. The locations and regions represented in the daily weather dataset were identical to those in the crop disaster dataset.

### Data cleaning

Unusual values in the collected data were corrected or excluded. The unusual values only occur in weather data and are defined as the value outside the range of the measurement. For example, the relative humidity is lower than zero. The range of variable was determined by the information of historical high and low values of the variable. After preprocessing, the two datasets were cleaned on the basis of different criteria such as missing proportion and outlier. In the crop disaster dataset, data obtained from the outlying islands were excluded because of their low frequency. Records for damaged field area with values smaller than 5 ha were excluded to avoid obtaining data representing the condition of only few fields. Disaster records that were not climatological or meteorological disasters, such as records of pest infestations and earthquakes, were excluded. Records from rare disasters, such as foehn winds, frost, hail, extremely high temperatures, and tornados, were also excluded. In addition, records of disasters with vague descriptions, such as unusual winds or unusual climate, were excluded. Moreover, records pertaining to vaguely identified crops, such as other coarse grains or special crops, were excluded.

Mean, maximum, and minimum air temperature; average relative humidity; total radiation; total sunshine hours; and total evaporation values lying >1.5 interquartile ranges (IQRs) below or above the first or third quartile, respectively, were considered outliers and excluded from the daily weather dataset. If more than 10 days of data (mean, maximum, or minimum air temperature; mean wind speed or direction; or total precipitation) were missing from a specific month, all data from that month were excluded. To ensure that the remaining data reflected the cultivation environment of most crops in Taiwan, data collected from weather stations with an altitude exceeding 1,200 m were excluded.

## Data Records

Table 2 presents the basic descriptions of the crop disaster and daily weather datasets, including their different spatial and temporal resolutions. The crop disaster dataset had a county-level spatial resolution and disaster-based temporal resolution. The daily weather dataset had a station-level spatial resolution and daily temporal resolution. The raw and clean datasets a were stored in the Figshare repository (10.6084/m9.figshare.c.6989844.v1)^22^. The files were named as follows: crop_disaster_raw.csv, crop_disaster_cleaned.csv, weather_raw.csv, and weather_cleaned.csv.

**Table 2:** Basic descriptions of crop disaster and daily weather datasets.

Dataset	Source	Spatial resolution	Temporal resolution	Format
**Crop disaster dataset**	MoA	county	disaster based	.csv
**Daily weather dataset**	CWA	station	daily	.csv

Table 3 lists the variables of the crop disaster dataset and their respective column headers. Because some variables were new additions or modified versions of older other variables, a version variable was used to distinguish original, new, and adjusted variables. Original variables were those from the Report. Adjusted variables were those that are modifications of original variables, such as estimated value loss after currency conversion from NTD into USD and variables reflecting the combination of city and county data. New variables were completely new additions to the dataset. In the crop disaster dataset, damage records for various crops in different counties could correspond to one disaster. After data cleaning, 233 crop disaster observations remained, which yielded 9,245 damage records in the dataset. The most common disaster in the dataset was typhoons (29.18%), and 62.20% of damage records related to typhoons. Seventy-four distinct crops were recorded in the crop disaster dataset. Rice (*Oryza sativa* L.) was the crop with the most damage records (576) in the dataset.

**Table 3:** List of variables in the crop disaster dataset and their respective headers.

Variables	Headers	Version
Disaster number	DISASTER_NO	New
Crop disaster 1	CROP_DISASTER1	Original
Crop disaster 2	CROP_DISASTER2	Original
Group of disaster	DISASTER_GROUP	New
Sub-group of disaster 1	SUB_DISASTER_GROUP1	New
Sub-group of disaster 2	SUB_DISASTER_GROUP2	New
Main type of disaster 1	DISASTER_MAIN_TYPE1	New
Main type of disaster 2	DISASTER_MAIN_TYPE2	New
Sub-type of disaster 1	DISASTER_SUB_TYPE1	New
Sub-type of disaster 2	DISASTER_SUB_TYPE2	New
Sub-sub-type of disaster 1	DISASTER_SUB_SUB_TYPE1	New
Sub-sub-type of disaster 2	DISASTER_SUB_SUB_TYPE2	New
Name of typhoon	EVENT_NAME	Original
Disaster occurrence year	YEAR	Adjusted
Disaster start date	START	Adjusted
Disaster end date	END	Adjusted
Affected location	COUNTY	Adjusted
Region	REGION	New
Type of crop	CROP_TYPE	New
Group of crop	CROP_GROUP	New
Class of crop	CROP_CLASS	New
Damaged crop	CROP	Original
Botanical name of crop	BOTANICAL_NAME	New
Damaged field area	FIELD_AREA	Original
Damaged level	DAMAGED_PERCENTAGE	Original
Actual damaged area	DAMAGED_AREA	Original
Estimated production loss	EST_LOSS_Q	Original
Estimated value loss	EST_LOSS_V	Adjusted

Table 4 lists the variables of the daily weather dataset and their respective column headers. In this dataset, a blank was used to indicate missing data. Compared with the other weather variables, the data for total sunshine hours, total radiation, and total evaporation were more frequently missing because most weather stations do not collect these data (Table 5). Data cleaning was therefore performed to reduce the proportion of records with missing weather data. Notably, none of the automatic rain gauge stations collect data on mean, maximum, or minimum air temperature or mean wind speed or direction. Therefore, while calculating the proportion of values missing for these variables in the clean dataset, we excluded data recorded by automatic rain gauge stations. The results indicated that 0.36%, 1.3%, 1.31%, 0.32%, 0.39%, and 0.54% of the possible mean air temperature, maximum air temperature, minimum air temperature, mean wind speed, mean wind direction, and total precipitation data, respectively, were missing from the clean dataset. Although much of the total sunshine hours, radiation, and evaporation data were missing, these variables were retained in the daily weather dataset because they are crucial to predicting crop growth.

**Table 4:** Daily weather dataset variables and their respective headers.

Variables	Headers	Version
Location of station	COUNTY	Adjusted
Region	REGION	New
Station code	SCode	New
Altitude of station	Altitude	New
Longitude of station	Longitude	New
Latitude of station	Latitude	New
Record date	Date	Original
Mean air temperature	meanT	Original
Maximum air temperature	maxT	Original
Minimum air temperature	minT	Original
Average relative humidity	RH	Original
Mean wind speed	meanWS	Original
Mean wind direction	meanWD	Original
Total precipitation	PREC	Original
Total radiation	RAD	Original
Total sunshine hours	SSH	Original
Total evaporation	EVAP	Original

**Table 5:** Number of data records and proportion of data missing from raw and cleaned daily weather datasets.

Variable	Raw		Cleaned	
	Data records (n)	Missing proportion (%)	Data records (n)	Missing proportion (%)
**meanT**	2,222,127	0.97	1,818,348	0.36
**maxT**	2,017,756	10.08	1,801,176	1.30
**minT**	2,017,292	10.1	1,801,105	1.31
**RH**	1,739,914	22.46	1,554,466	14.82
**meanWS**	2,195,673	2.15	1,819,063	0.32
**meanWD**	2,197,768	2.06	1,817,785	0.39
**PREC**	3,562,536	1.23	289,9734	0.54
**SSH**	582,861	74.02	336,228	81.58
**RAD**	405,746	81.92	347,608	80.95
**EVAP**	282,636	87.4	224,709	87.69

## Technical Validation

Technical validation was initially conducted by comparing the EM-DAT and GLIDE datasets. Descriptive statistics for weather and impact variables were used to validate the reliability and variation of the measurement values.

### Comparison with other disaster databases

**Fig. 1:**
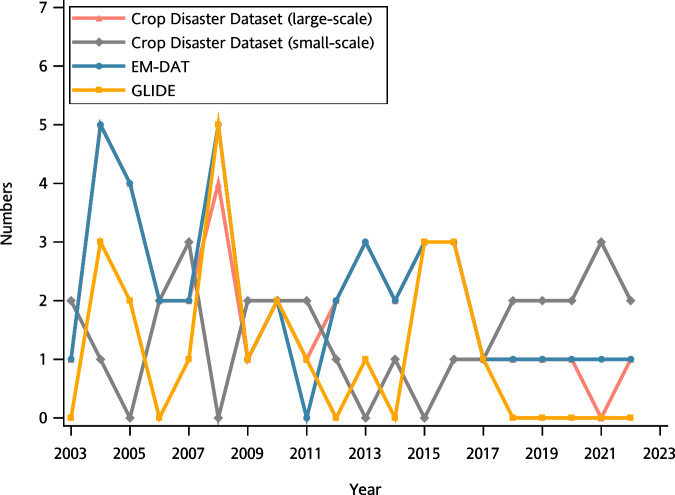
Annual tropical storm disasters recorded in the crop disaster dataset, EM-DAT database, and GLIDE database.

Overall, the primary contribution of this study lies in providing information regarding the effects of disasters, particularly small-scale and silent disasters, on crops. To assess the validity of the purposes, we compared the numbers of disasters included in the EM-DAT and GLIDE databases. Unlike our crop disaster dataset, these two national-scale databases primarily include large-scale disasters. Because of the low enrollment rate of other disasters in Taiwan in the EM-DAT and GLIDE databases, we specifically compared the numbers of tropical storms. Data on 69, 41, and 23 tropical storms from 2003 to 2022 appeared in the crop disaster dataset, EM-DAT database, and GLIDE database, respectively. The match ratio^23^ was calculated to determine whether the tropical storm data in the crop disaster dataset coincide with the data in the other databases. The name of each tropical storm and the year of its occurrence were used to match the tropical storms in each database. The match ratio, which was calculated as 0.93, was defined as the proportion of tropical storms in the EM-DAT or GLIDE databases recorded in the crop disaster dataset. The mismatch between crop disaster dataset and two disaster databases was caused by tropical storm Hagupit (2008), Goni (2015), and Choi-Wan (2021). Two of these tropical storms (Hagupit and Goni) did not make landfall in Taiwan. Choi-Wan weakened into a tropical depression while its radius still arrived in southern Taiwan. Therefore, these three tropical storms were not recorded in the crop disaster dataset. As shown in Fig. 1, the numbers of large-scale tropical storms differed among the datasets and fluctuated every year. However, the fluctuation patterns were similar, indicating similar trends. With the exception of 2008, the number of tropical storms in the crop disaster dataset was equal to or greater than those in the other two databases. In addition, the yearly tropical number between crop disaster dataset and EM-DAT is identical in most of the years. The differences between crop disaster dataset and EM-DAT were observed in the years of 2008, 2011, and 2021. The differences in 2008 and 2021 resulted from the reason that Hagupit and Choi-Wan were not recorded as the tropical storm disaster in the crop disaster dataset. The difference in 2011 was tropical storm Nanmadol which was recorded in both crop disaster dataset and GLIDE, but it was not recorded in EM-DAT. All tropical storms that were not recorded in the EM-DAT and GLIDE databases were regarded as small-scale disasters. The frequency of small-scale tropical storms also displayed a fluctuation from 2003 to 2022. However, a notable increasing frequency was observed between 2015 and 2022.

Impact variables were used to present the scale of disasters. Tropical storms in the crop disaster dataset were divided into large-scale and small-scale storms. In total, 29 out of 69 tropical storms were small-scale disasters (Table 6). Clear differences were observed in the damaged field area, actual damaged area, estimated production loss, and estimated value loss of large-scale and small-scale disasters. Although the difference in damage level between small-scale and large-scale disasters was not as large as those in other impact variables, large-scale tropical storms still resulted in greater damage level. These results indicate that the crop disaster dataset contains a mostly complete record of tropical storms and includes both small-scale and large-scale disasters.

**Table 6:** Mean values of impact variables of small-scale and large-scale tropical storms in the crop disaster dataset.

Disaster	Disaster Numbers	Damaged Field Area (ha)	Damaged Level (%)	Actual Damaged Area (ha)	Estimated Production Loss (tonne)	Estimated Value Loss (US$ 1,000)
Small-scale	29	1,811.17	21.5169	360.49	4,824.93	4,019.50
Large-scale	40	26,814.76	25.0275	7,484.81	95,250.04	76,940.78

### Measurement value variations

The mean, standard deviation (STD), maximum (Max), minimum (Min), and IQR of the weather and impact variables were used to examine the reliability and variation of the measurement values. After the data had been cleaned, the maximum of maximum air temperature (maxT), average relative humidity (RH), mean wind speed (meanWS), total radiation (RAD), and total evaporation (EVAP) were calculated as 40.1, 100, 33.6, 35.04, and 8.5, respectively (Table 7). The maximum PREC caused by the 2009 Typhoon Morakot was 1190. In terms of weather variables, a minimum of mean wind direction (meanWD) value of 0 indicated the absence of wind (wind speed <0.2 m s^−1^). A negative EVAP value indicated the occurrence of rainfall during the measurement period.

**Table 7:** Mean, standard deviation (STD), maximum (Max), minimum (Min), and interquartile range (IQR) of weather variables in the cleaned daily weather dataset.

Variables	Mean	STD	Max	Min	IQR
meanT	22.91	4.96	34.8	5.6	7.80
maxT	27.26	5.50	40.1	9.5	8.20
minT	19.81	4.93	31.8	3	7.70
RH	81.69	9.56	100	54	14
meanWS	1.76	1.52	33.6	0	1.4
meanWD	131.89	117.06	360	0	206
PREC	6.37	23.29	1190.00	0	2
SSH	5.63	3.77	13.1	0	7
RAD	13.21	7.1	35.04	0	10.91
EVAP	2.66	2.07	8.5	−3.3	3

The descriptive statistics of the impact variables for each disaster were calculated in the crop disaster dataset. A single disaster could affect more than one county, and a large-scale disaster would be expected to affect multiple counties. After the disasters were divided into four scales depending on the number of affected counties, statistical calculations were conducted. The number of affected counties corresponding to scales 1, 2, 3, and 4 was 1, 2–3, 4–8, and >8, respectively. As expected, when the scale of a disaster was greater, the mean values of the impact variables were also greater except for the level of damage (Table 8). Compared with the other impact variables, the mean values of the level of damage were more stable. Among the potential reasons underlying the great variations observed in the damaged field area, actual damaged area, estimated production loss, and estimated value loss were the disaster type, affected county, disaster occurrence timing, and damaged crop type. After the data had been cleaned, the minimum values of damaged field area, damage level, actual damaged area, estimated production loss, and estimated value loss were calculated as 5, 5, 0.25, 2.4, and 1.36, respectively, in the crop disaster dataset. The maximum damaged field area, actual damaged area, estimated production loss, and estimated value loss were observed in the record of the 2016 Typhoon Megi. The highest level of damage in the crop disaster dataset, namely 54.67, was caused by the rain disaster of 2022. In most of the impact variables, the IQR exhibited smaller variation than the STD did.

**Table 8:** Mean, standard deviation (STD), maximum (Max), minimum (Min), and interquartile range (IQR) of impact variables in the cleaned crop disaster dataset at different scales.

Variables	Scale	Mean	STD	Max	Min	IQR
**Damaged Field Area**	1	216.18	480.93	2,897.13	5.00	165.72
	2	811.78	1,179.64	6,798.27	15.00	716.16
	3	3,155.63	4,138.51	21,900.98	95.39	2,344.11
	4	25,913.29	31,050.72	137,467.92	1,548.97	28,851.65
**Damaged Level**	1	26.49	11.58	54.67	5	15.03
	2	26.21	10.86	54.8	5	14.49
	3	24.83	8.03	44.25	11.26	12.89
	4	24.51	5.88	40.43	11.54	6.33
**Actual Damaged Area**	1	69.24	182.60	1,124.00	0.25	45.24
	2	245.11	457.23	2,809.07	0.75	164.62
	3	866.49	1,235.20	6,118.44	13.00	673.07
	4	7,156.73	9,123.74	42,465.13	243.89	8,296.28
**Estimated Production Loss**	1	979.55	2,732.01	18,699.00	2.40	700.32
	2	2,865.02	4,909.69	26,887.65	16.53	2,639.16
	3	10,438.04	17,032.23	99,776.73	153.58	10,235.70
	4	87,462.07	107,377.17	488,729.91	2,749.30	85,249.21
**Estimated Value Loss**	1	1,135.11	3,485.73	22,138.25	1.36	624.35
	2	4,226.97	10,772.05	74,194.43	19.88	3,135.64
	3	10,299.65	14,976.70	73,237.78	184.37	8,744.71
	4	79,054.60	90,148.00	465,717.10	2,697.13	86,521.61

## Usage Notes

Both the raw and the clean versions of each dataset were uploaded to an online repository (10.6084/m9.figshare.c.6989844.v1) (for more details, please refer to the Methods section). To link the two datasets, affected locations, disaster start dates, and disaster end dates were obtained from the crop disaster dataset and matched to the location of each station and record date in the daily weather dataset. Overall, the datasets described in this study can be publicly accessed. They can also be used without restrictions except for a citation of this data descriptor article and the dataset used.

## Data Availability

Most of the weather data used in this study were downloaded using a Python script. Only weather data obtained from agricultural weather stations were manually downloaded. All datasets were processed and analyzed using SAS. The Python and SAS codes are available at https://github.com/YuanChihSu/Crop_Disaster_Dataset. A full list of weather station codes and altitudes is also provided.
